# Administration Routes for Perioperative Prophylactic Antibiotics: A Scoping Review of Intravenous Push Versus Infusion

**DOI:** 10.3390/antibiotics15070643

**Published:** 2026-06-27

**Authors:** Canyu Yang, Shuhua Deng, Yuan Wei, Yuxi Xia, Xiaoning Yuan, Ning Shen, Li Yang, Rongsheng Zhao, Suodi Zhai, Yingqiu Ying

**Affiliations:** 1 Department of Pharmacy, Peking University Third Hospital, Beijing 100191, China; ycy0414@bjmu.edu.cn (C.Y.); lilianyangli@163.com (L.Y.); zhaorongsheng@bjmu.edu.cn (R.Z.); zhaisuodi@163.com (S.Z.); 2 Department of Operating Room, Peking University Third Hospital, Beijing 100191, China; weishei@163.com; 3 Department of Obstetrics and Gynecology, Peking University Third Hospital, Beijing 100191, China; weiyuanbysy@163.com; 4 Department of Medical Administration, Peking University Third Hospital, Beijing 100191, China; 15810866570@163.com; 5 Department of Hospital Infection Control, Peking University Third Hospital, Beijing 100191, China; yxnsby@163.com; 6 Department of Respiratory and Critical Care Medicine, Peking University Third Hospital, Beijing 100191, China; puh3shenning@bjmu.edu.cn

**Keywords:** surgical antibiotic prophylaxis, intravenous push, intravenous infusion, surgical site infection, pharmacokinetics/pharmacodynamics, scoping review, clinical pharmacist

## Abstract

Objectives: Surgical site infections (SSIs) represent a significant postoperative challenge. Although timely perioperative prophylaxis with cephalosporins is essential to prevention, adherence to the recommended 30–60 min administration window may be challenging with traditional intravenous infusion (IVI) in settings with high surgical turnover, as is the case in China. Intravenous push (IVP) has been proposed as a more time-efficient alternative. This scoping review aims to map the available evidence comparing IVP with IVI for perioperative cephalosporin administration across four domains: safety, pharmacokinetics/pharmacodynamics (PK/PD), efficacy, and economic impact. Methods: A systematic search was conducted across PubMed, Embase, Web of Science, the Cochrane Library, and gray literature up to February 2026. Data were systematically charted and extracted using a standardized form. Results: Of the 14 included sources, only 3 were peer-reviewed comparative studies; the remaining 11 (78.6%) were gray literature documents. Among the gray literature, 72.7% (8/11) permitted or recommended IVP for cephalosporin prophylaxis; however, this proportion reflected practice patterns of heterogeneous methodological rigor. The 3 peer-reviewed studies focused on the safety, PK/PD, and economic outcomes. Two studies—in orthopedic and bariatric surgery, respectively—found no significant difference in adverse event rates between IVP and IVI, though both were limited by small samples. A single small study suggested similar PK/PD target attainment between IVI and IVP cefazolin. No study directly compared SSI rates between the two routes. One study suggested potential cost savings with IVP, but the evidence was dated and based on limited patient numbers. Conclusions: The available evidence for IVP is predominantly derived from gray literature, while peer-reviewed articles suggest that safety and PK/PD profiles do not differ markedly from IVI in the limited populations, surgical procedures, and agents studied; economic data are suggestive but dated. Direct comparative data on clinical efficacy outcomes, such as SSI rates, are absent. Well-powered, multi-center comparative studies comparing IVP and IVI with SSI as a primary endpoint are needed.

## 1. Introduction

Surgical site infections (SSIs) constitute a major global public health concern, consistently ranking among the most common postoperative complications [1,2]. They significantly prolong hospital stays, increase readmission rates, escalate healthcare costs, and are directly linked to adverse long-term patient outcomes [3]. Consequently, appropriate and timely perioperative prophylactic antibiotic administration is critical to ensure adequate drug concentrations at the surgical site, thereby reducing the risk of postoperative infections [4].

Beta-lactam antibiotics, notably first- and second-generation cephalosporins such as cefazolin and cefuroxime, are the most widely recommended agents for perioperative prophylaxis due to their broad-spectrum activity, favorable safety profile, and reliable coverage of common surgical pathogens [5]. As time-dependent agents, their efficacy is closely associated with maintaining the free drug concentration above the minimum inhibitory concentration (fT > MIC) for a sufficient proportion of the dosing interval. This pharmacodynamic property necessitates not only the correct choice of agent but also an optimized administration method to ensure that therapeutic thresholds are reached before the first surgical cut [6].

Most international clinical guidelines [7,8] recommend that the preoperative antibiotic dose be completed within 60 min prior to skin incision to ensure adequate tissue concentrations. However, the requirements are often stricter in Chinese clinical guidelines, which specifically mandate administration within the 30 to 60 min pre-incision window [9]. Achieving this strict timing is frequently suboptimal in real-world surgical settings. Because the intravenous infusion (IVI) remains the predominant administration route, and the inherent infusion time of standard prophylactic agents like cefazolin and cefuroxime (often requiring 30 min or more) presents a barrier for timely completion.

Studies have shown that the median interval from patient entry into the operating room to surgical incision ranges from 21 to 49 min [10]. Within this condensed timeframe, complex preparatory tasks such as anesthesia induction, positioning, and draping often leave insufficient time for a complete IVI to finish before surgery begins. Consequently, patients may receive potentially inadequate antibiotic exposure at the critical moment of incision. Another study from the US demonstrated that the average start time for cefazolin was only 19.66 ± 15.78 min before incision [11]. Consequently, incomplete IV infusion may lead to subtherapeutic plasma concentrations and insufficient tissue exposure at the critical moment of incision.

IVP administration has been proposed as a rapid, time-efficient alternative. By delivering the medication over 3 to 5 min, IVP may enable target plasma concentrations to be achieved more promptly, potentially mitigating the procedural delays inherent to IVI. Although IVP is indicated on the drug labels for several key beta-lactam antibiotics (e.g., cefazolin, cefuroxime, ceftriaxone), its wider adoption for perioperative prophylaxis has been limited, especially in settings like China, possibly due to a perceived lack of comprehensive data. Importantly, the integrated comparative evidence, especially regarding safety, PK/PD profiles, clinical efficacy (SSI rates), and economic impact of IVP versus IVI in the perioperative setting, remains fragmented across diverse sources, including clinical trials and institutional guidelines.

Therefore, this scoping review aims to systematically map the literature comparing IVP and IVI for perioperative antibiotic prophylaxis. Using the Population, Concept, and Context (PCC) framework, we intend to evaluate the evidence across four critical dimensions: safety, PK/PD profiles, clinical efficacy, and economic considerations. By synthesizing this evidence, this review seeks to provide a foundation for evidence-based clinical protocols and identify specific knowledge gaps to guide future high-quality research in surgical antimicrobial stewardship.

## 2. Methods

All aspects of the five-step methodology proposed by the Joanna Briggs Institute (JBI) guidance were followed, and the study was reported according to the Preferred Reporting Items for Systematic Reviews and Meta-Analyses extension for Scoping Reviews (PRISMA-ScR) guidelines [12].

### 2.1. Data Sources and Search Strategy

A comprehensive systematic search was performed from database inception to February 2026 across the PubMed, Embase, Web of Science, and the Cochrane Library databases. The main terms included “surgical antibiotic prophylaxis,” “intravenous push,” and “intravenous infusion.” To ensure the inclusion of current clinical standards, an extensive search for gray literature was also performed via Google and the official websites of major health organizations using the search string “surgical antibiotic prophylaxis administration guideline OR practice.” To ensure adequate coverage, the first 150 relevance-ranked records returned by each search string were manually screened. Targeted gray literature primarily included clinical practice guidelines and hospital internal institutional protocols. No date restrictions were applied. Detailed search strings for each database are provided as Supplementary Data.

### 2.2. Eligibility Criteria

The selection of literature followed the PCC (Population, Concept, Context) framework:

- Population: Adult patients (≥18 years) undergoing surgical procedures requiring perioperative antibiotic prophylaxis.
- Concept: Comparative evidence between IVP and IVI methods for a single preoperative dose.
- Context: The perioperative setting, focusing on administration timing, safety, PK/PD attainment, clinical efficacy and economic impact.

We included peer-reviewed original research (RCTs, observational studies) and clinical practice guidelines/protocols. Exclusion criteria were studies focusing on pediatric populations, non-prophylactic use, and case reports, and articles where the full text was unavailable.

Institutional guidelines and clinical protocols published by major academic medical centers, national health agencies, or specialized professional organizations were considered gray literature. The inclusion of these documents is essential to address the implementation gap regarding real-world methods. Documents were eligible if they were issued by an authoritative body and specified the intravenous administration method (IVP or IVI) for the prophylactic agent.

### 2.3. Study Selection

**Figure 1:**
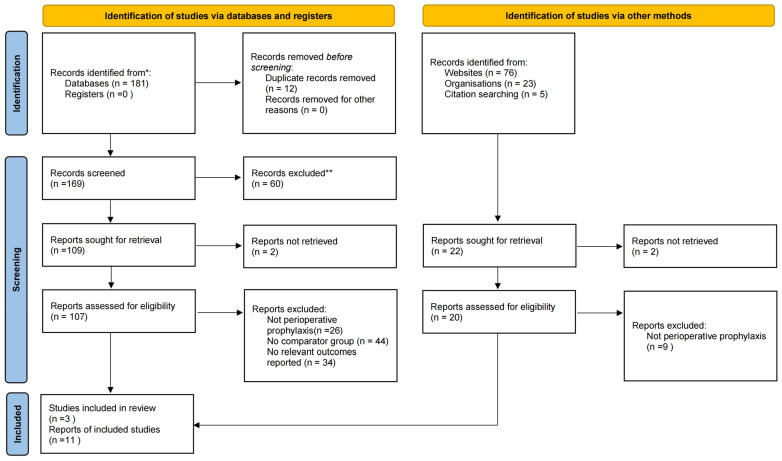
PRISMA 2020 flow diagram showing the selection process for included studies.

References were collected in Zotero, and duplicates were removed. Two reviewers (C.Y. and Y.Y.) independently screened the titles and abstracts identified in the search. In case of disagreement, a consensus was reached through discussion or by consulting a third reviewer. After initial screening, potentially relevant articles were included for full-text screening. A PRISMA flow diagram provides an overview of the selection process and the number of sources retrieved and excluded at each stage (Figure 1).

### 2.4. Data Extraction and Synthesis

For included articles and gray literature, standardized data charting forms were developed to record key information. For gray literature, antibiotic agents, doses, and specific administration time/duration mandated were extracted. For included articles, the author, year, sample size, groups, and outcomes of interest were extracted. Given the nature of a scoping review to map the available evidence rather than assess individual study quality for meta-analysis, the findings were synthesized descriptively to provide a comprehensive map of the evidence landscape.

## 3. Results

### 3.1. Overview of Included Sources

A total of 14 documents met the predefined eligibility criteria. In accordance with the PCC framework guiding this review, all included sources addressed adult surgical patients receiving perioperative antibiotic prophylaxis (Population), provided information on the comparison between IVP and IVI administration (Concept), and were situated within perioperative care settings (Context). Consistent with the scarcity of comparative evidence, the vast majority were gray literature [13,14,15,16,17,18,19,20,21,22,23] (*n* = 11), while only three were peer-reviewed articles retrieved via systematic database searches. The three academic articles included: one randomized controlled trial (RCT), one prospective cohort study, and one comparative PK study [24,25,26]. These were published between 1992 and 2012, highlighting a lack of contemporary high-quality evidence over the past decade, despite the increasing adoption of IVP in clinical practice. Studies were primarily focused on cefazolin (*n* = 2) and cefmetazole (*n* = 1), and surgical settings were orthopedic surgery (*n* = 1) and bariatric surgery (*n* = 1), and one study did not specify a detailed surgical procedure. Two studies reported on safety (one of which also reported economic outcomes), one on PK/PD, and one on efficacy-related surrogate endpoints.

A total of 11 documents were identified as gray literature (Table 1), consisting of institutional (*n* = 5) and national/regional (*n* = 6) clinical practice guidelines and protocols. The national and regional documents represented a diverse range of healthcare jurisdictions, including organizations from Scotland (SIGN, SAPG), the United States (ASHP/IDSA), Australia, and the United Kingdom (NICE). The institutional protocols were derived from leading academic medical centers and health systems, such as UCSF Medical Center, Johns Hopkins University, and Peking Union Medical College Hospital (China), reflecting localized clinical standards and operational workflows. The publication years spanned over two decades, from 2000 to 2024. The majority of the documents (*n* = 7, 63.6%) were published or updated within the last five years (2020–2024). Only one foundational guideline (SIGN 45) dated back to 2000, while the remaining sources were distributed between 2013 and 2018 (*n* = 3, 27.3%). This distribution provides a comprehensive overview of the evolution and current state of administration route recommendations for surgical prophylaxis.

However, the methodological basis of these 11 documents spans a wide spectrum. Several institutional protocols were produced through internal consensus without transparent reporting of evidence sources or formal appraisal of the comparative evidence for IVP versus IVI. Institutional documents may also be shaped by context-specific factors rather than by demonstrated clinical equivalence.

### 3.2. Administration Route Recommendations in Gray Literature

Among the 11 gray literature documents, a majority (8 of 11, 72.7%) recommended or permitted IVP for the initial prophylactic cephalosporin dose. This proportion should be interpreted cautiously, given the substantial heterogeneity in methodological rigor, evidentiary basis, geographic origin, and clinical setting across the included documents; in particular, institutional protocols may reflect local operational considerations rather than formal comparative evidence assessments. Three documents (27.3%) presented non-specific or ambiguous instructions regarding the administration route. As shown in Table 2, Cefazolin, as the primary reference agent (7 of 11 sources), was consistently recommended for IVP administration over 3–5 min, with standard dosing of 2 g and weight-adjusted dosing of 3 g for patients ≥100–120 kg. Cefuroxime (1.5 g) and ceftriaxone (1–2 g) were also recommended for IVP over 2–5 min by 3 sources each. Cefmetazole, cefoxitin, and cefepime each had single-source IVP recommendations. Preparation details, including dilution volumes and concentrations, were provided by only two sources [13,16] and were limited to specific antibiotics.

The remaining 3 documents (27.3%) presented non-specific or ambiguous instructions. Specifically, two documents [14,23] vaguely instructed ‘IV administration’ without specifying the technique or duration, while one [21] lacks any explicit information regarding the preferred route (IVP vs. IVI) or the required duration.

### 3.3. Comparative Evidence on Safety Outcomes

Two studies compared the safety of IVP with IVI in the context of perioperative prophylaxis against surgical site infections (SSIs). As shown in Table 3, one study [25] enrolled 240 patients undergoing orthopedic surgery to evaluate the safety of cefazolin administered via IVP or IVI. In the IVP group, 1–2 g of cefazolin was dissolved in 10–20 mL of normal saline and administered over a period of 3–5 min or 6–10 min. The incidence of phlebitis was comparable between the two groups (3.3% with IVP vs. 3.4% with IVI). Similarly, a prospective randomized trial [26] involving 60 patients compared IVP and IVI administration of cefmetazole for perioperative prophylaxis. No adverse events were observed in either group, and the incidence of phlebitis did not differ significantly.

### 3.4. Comparative Evidence on PK Outcomes

The PK characteristics of IVP vs. IVI were also compared in perioperative surgical infection prophylaxis (Table 4). One study [24] compared a 5 min IVP and a 30 min IVI of 2 g of cefazolin in morbidly obese patients undergoing gastric bypass surgery. Plasma concentration–time profiles at 30, 120, and 360 min post-administration were not significantly different between the two groups. The C_30min_ was 140.6 ± 62.9 μg/mL for IVP and 165.1 ± 38.5 μg/mL for IVI, respectively. The elimination half-life of cefazolin was 2.3 ± 0.4 h with IVP and 2.9 ± 1.1 h with IVI. The findings suggest that IVP may achieve a comparable PK profile. However, the sample size was small (*n* = 15) and limited to the specific population and the single surgical procedure.

### 3.5. Comparative Evidence on Efficacy Outcomes

Due to the limited number of included clinical studies, no direct efficacy outcomes, such as SSI rates, were available. Therefore, the only available efficacy-related data came from the same study [24] in morbidly obese patients, which reported the pharmacodynamic endpoint fT > MIC as a surrogate marker for clinical efficacy (Table 5). Using an MIC of 8 μg/mL, the calculated fT > MIC was 3.6 h for IVP versus 3.4 h for IVI, and the calculated protective duration was 5.1 h (IVP) and 4.8 h (IVI), respectively. This suggested that the rapid IVP administration method may not substantially compromise the expected duration of prophylactic drug exposure above the critical threshold, although the small observed difference (0.2 h) is of uncertain clinical significance. These surrogate endpoints derive from a single study of 15 patients and have not been validated against SSI outcomes.

### 3.6. Comparative Evidence on Economic Outcomes

One study [26] assessed the economic implications of IVP vs. IVI (Table 6). IVP administration resulted in a direct cost saving of $3.25 per dose due to the reduced need for infusion-related materials (e.g., bags, tubing). The study also highlighted operational efficiencies, estimating a labor cost saving of $0.60 per dose from reduced preparation and administration time, projecting an annual institutional saving of $184,000 across both prophylactic and therapeutic use. These findings, while informative, reflect healthcare economics from over three decades ago (1992), and their applicability to current clinical practice is limited.

### 3.7. Knowledge Gaps Identified by Evidence Mapping

The evidence mapping process identified several knowledge gaps. Firstly, comparative studies were limited to first- and second-generation cephalosporins. Secondly, the evidence base is only derived from orthopedic and bariatric surgery, which lack high-volume procedures like general surgery, obstetrics/gynecology, limiting extrapolation. Thirdly, there is a critical absence of direct comparative data on meaningful clinical efficacy outcomes such as SSI rates, and current literature relies on surrogate markers such as PK/PD indices (fT > MIC), but their relationship to actual SSI prevention has not been validated in this context. These gaps underscore the need for adequately powered RCTs with SSI as a primary endpoint to inform guidelines beyond the currently studied surgical and antibiotic settings.

## 4. Discussion

The timely administration of perioperative antibiotic prophylaxis is widely regarded as one of the fundamental factors in minimizing the risk of SSIs. While Chinese guidelines recommend completion of the dose 30–60 min prior to incision [27], the inherent duration of traditional IVI may pose challenges in adhering to this narrow window, particularly in high-volume surgical centers where theater turnover is rapid.

Through the lens of the PCC (Population, Concept, Context) framework, this scoping review mapped the evidence comparing IVP and IVI for perioperative cephalosporin prophylaxis in adult surgical patients across heterogeneous clinical settings. A central finding of this review is the marked imbalance in the evidence base: of 14 included sources, 11 (78.6%) were gray literature documents, while only 3 peer-reviewed comparative studies were identified. This imbalance carries important implications for interpretation, as conclusions drawn predominantly from guidelines and institutional protocols—which vary in methodological rigor—cannot be accorded the same weight as those derived from well-designed clinical studies.

Our evidence mapping of clinical practice guidelines (gray literature) suggested that over 70% (8/11) of included documents permitted IVP for cephalosporin administration, indicating a degree of clinical familiarity with this administration route. However, this proportion should not be equated with high-level evidence of comparative effectiveness or safety. Many of these documents, especially institutional protocols, may reflect local pragmatic considerations, such as nursing workflow efficiency or resource constraints, rather than evidence-based assessments of IVP versus IVI. The high prevalence of IVP endorsement therefore reflects clinical acceptability rather than a consensus evidence-based recommendation.

The peer-reviewed evidence is confined to three studies published between 1992 and 2012 (total *n* = 15–240 patients). Regarding safety, data drawn from two studies in orthopedic and bariatric surgery indicated that standardized IVP may be well tolerated, with no statistically significant difference in local adverse reaction rates compared with IVI in the populations and agents studied. PK/PD data from a single study of 15 morbidly obese patients indicated similar PK profiles and fT > MIC target attainment between the two routes. Eelsing et al. [28] also demonstrated in a prospective cohort that a 2 g prophylactic cefazolin by IVP achieves concentrations in plasma and target-site tissues during lower extremity surgery that appeared adequate in the studied cohort. By confirming that tissue concentrations remain significantly above the MIC_90_ for at least 80 min, these data suggest that the IVP may provide prompt and potentially adequate antimicrobial coverage at the surgical incision. However, the relationship between plasma PK/PD surrogates and actual tissue concentrations at the surgical site and SSI rate has not been directly validated. Nevertheless, while these PK/PD data are reassuring, they cannot substitute for direct comparisons of SSI rates, which remain the definitive clinical endpoint. No included study directly compared SSI rates between IVP and IVI, and this gap remains the most important limitation of the evidence base. Several additional limitations constrain the generalizability of these findings. The peer-reviewed studies are limited to two surgical populations (orthopedic and bariatric surgery) and two antibiotics (cefazolin and cefmetazole); extrapolation to other surgical specialties or prophylactic agents is not supported by the current evidence. Furthermore, the risk–benefit calculus for IVP versus IVI may differ by procedure type. In specialties with low baseline SSI rates, the logistical advantage of IVP may be less compelling, whereas in high-risk, high-volume procedures the time-efficiency gains may carry greater clinical weight—though neither scenario has been directly studied. All three studies were small, and the economic data derive from a single study published over 30 years ago, offering limited guidance for contemporary practice.

Beyond the perioperative setting, the safety and efficacy of IVP are further supported by observations in emergency and critical care medicine. Retrospective clinical evaluations indicate that the safety profile of IVP administration for select β-lactams tends to be comparable to that of IVI. In a large-scale review of 1000 patients receiving IVP aztreonam, ceftriaxone, cefepime, or meropenem, the observed incidence of adverse drug events (ADEs) was only 1% [29], with allergic reactions and neurotoxicity occurring at rates similar to historical IVI data. Furthermore, it was also reported that clinical outcomes such as 28-day mortality in septic patients and microbiological clearance in Gram-negative bacteremia have not shown significant variation (HR 1.07; 95% CI 0.69–1.65) based on the administration route [30]. Similarly, in the management of Gram-negative bacteremia, IVP (5 min) and IVI (30 min) cefepime or meropenem achieved comparable microbiological clearance rates and clinical response [31]. However, these findings from therapeutic settings should be extrapolated to the prophylactic perioperative context with caution, as the patient populations and treatment objectives differ substantially.

Furthermore, the potential for resource optimization through IVP administration warrants consideration. Traditional IVI systems involve multiple single-use components, including infusion bags and extensive delivery tubing. In contrast, IVP streamlines the administration hardware to a single syringe. A review in 2019 [32] reported that the cost of cefazolin 1 g (IVP) was $0.75, while 2 g (IV piggyback) was $6.83, and the difference in costs could be significant in large centers using cefazolin prophylaxis for cardiothoracic, orthopedic, obstetrics/gynecology, and bariatric surgery.

However, regulatory labeling represents a structural determinant of IVP adoption. In several jurisdictions, package inserts for commonly used cephalosporins do not include IVP as an approved route; for example, Chinese manufacturer instructions for cefazolin typically specify only intravenous infusion, effectively rendering IVP off-label. Under such constraints, IVI becomes the default, and clinical guidelines may not address IVP as a distinct route. This may explain why only one Chinese institutional protocol was identified [13], notably precisely because it explicitly permits IVP despite the prevailing labeling environment. Beyond labeling, the choice between IVP and IVI is also shaped by practitioner-level preferences, which diverge by professional role. A small survey [33] of anesthetists (*n* = 35) reported that 97% of them (34/35) preferred IVP cefazolin over IVI for perioperative antibiotic administration, and the main reasons for this choice were practicality and familiarity. Interestingly, 58% thought there was no difference in safety, 12% thought IVI was safer, while none of them thought IVP was safer. However, IVP places distinct demands on nursing staff. Unlike the passive monitoring of IVI, standardized IVP requires an active nursing presence for 3 to 5 min. In high-turnover operating rooms, rapid surgical turnover makes this dedicated time-lock difficult to maintain. Such engagement often conflicts with essential perioperative tasks like instrument preparation. Furthermore, frequent task interruptions in the operating theater increase the risk and severity of medication errors by 12.1% per occurrence [34]. Nurses may perceive the manual requirement of IVP as a cognitive burden during urgent intraoperative demands.

Several limitations of this review should be acknowledged. Firstly, the evidence base relies predominantly on gray literature of variable quality; secondly, no study included in this review used SSI as a primary endpoint to compare IVP and IVI. Therefore, the observation of similar PK/PD target attainment between the two routes suggests biological plausibility but does not constitute proof of clinical equivalence in SSI prevention. Lastly, the three peer-reviewed articles were all limited by small sample sizes, a narrow surgical and antibiotic scope, and dated economic analyses. Well-powered, multicenter comparative studies with SSI as a primary endpoint across diverse surgical populations are needed.

Considered within the PCC framework, the evidence landscape reveals that the Population dimension remains narrowly studied, the Concept of comparative IVP versus IVI evidence is predominantly informed by gray literature rather than controlled studies, and the Context spans heterogeneous healthcare systems whose regulatory and operational differences may influence IVP adoption independently of the comparative evidence. These gaps define the agenda for future research.

## 5. Conclusions

The present scoping review systematically maps the available evidence comparing IVP with IVI for perioperative antibiotic prophylaxis. While IVP is referenced in the majority of included clinical practice guidelines, the evidence is insufficient to support definitive conclusions regarding its comparative safety and efficacy, owing to the limited number of studies, small sample sizes and confinement of the peer-reviewed evidence to orthopedic and bariatric surgery with cefazolin or cefmetazole. No peer-reviewed study has directly compared SSI rates between IVP and IVI, and the relationship between available PK/PD surrogate endpoints and clinical outcomes has not been validated in the perioperative context. Well-powered, multicenter randomized trials with SSI as a primary endpoint are needed across broader surgical populations and prophylactic agents.

## Figures and Tables

**Table 1:** Overview of included gray literature documents.

Classification	Ref.	Issuing Body	Country	Year	IVP/IVI
National	[21]	NASEP, Singapore	Singapore	2022	NS ^#^
	[22]	Scottish Antimicrobial Prescribing Group (SAPG)	UK	2022	IVP
	[18]	SAAGAR, SA Health	Australia	2021	IVP
	[14]	ASHP/IDSA/SIS/SHEA	USA	2013	NS
	[23]	SIGN	UK	2000	NS
Regional	[20]	Alberta Health Services	Canada	2018	IVP
Institutional	[15]	Emory Healthcare	USA	2024	IVP
	[13]	Peking Union Medical College Hospital	China	2023	IVP
	[17]	UCSF Medical Center	USA	2021	IVP
	[19]	Johns Hopkins University	USA	2021	IVP
	[16]	UNC School of Medicine	USA	2017	IVP

^#^ NS: not specified, absence of specific IV-specific guidance, such as IVI and IVP, rather than opposition.

**Table 2:** Summary of IV administration route of antibiotics in SSI prevention.

Classification	Antibiotics	IVP	Dose	Duration	Preparation	Ref.
Access	Cefazolin	Yes	≤120 kg: 2 g >120 kg: 3 g	/	/	[15]
		Yes	2 g	Over 3–5 min	SWFI * (100 mg/mL)	[16]
		Yes	2 g	/	/	[20]
		Yes	40–120 kg: 2 g >120 kg: 3 g	Over 3–5 min	/	[17]
		Yes		Over 5 min	/	[18]
		Yes	/	/	/	[21]
		Yes	<100 kg: 2 g ≥100 kg: 3 g	Over 3–5 min	/	[19]
Watch	Cefuroxime	Yes	1.5 g	Over 3–5 min	1.5 g–12 mL SWFI	[13]
		Yes	1.5 g	Over 3–5 min	1.5 g–16 mL SWFI	[16]
		Yes	1.5 g	/	/	[20]
	Ceftriaxone	Yes	<40 kg: 1 g >40 kg: 2 g	Over 3–5 min		[17]
		Yes	1 g	/	/	[20]
		Yes	2 g	2–4 min	1 g–10 mL SWFI	[13]
	Cefmetazole	Yes	1–2 g	/	1 g–10 mL SWFI	[13]
	Cefoxitin	Yes	2 g	Over 3–5 min	2 g–10 or 20 mL SWFI	[16]
	Cefepime	Yes	2 g	Over 3–5 min	500 mg–5 mL SWFI	[16]

* SWFI: sterile water for injection. /: not mentioned.

**Table 3:** Safety outcomes of IVP and IVI in SSI prevention.

Author	Year	No.	Groups	Safety Outcomes	Ref.
Biggar, C. et al.	2012	240	IVP (*n* = 120): 2 g of cefazolin administered by IVP IVI (*n* = 120): 2 g of cefazolin administered by IVI	No significant difference in phlebitis rates between groups. IVP: Phlebitis rate: 3.3% (4/120). All 4 cases were grade 1. IVI: Phlebitis rate: 3.4% (4/118). Grade 1: 2 patients. Grade 2: 2 patients.	[25]
J C Garrelts et al.	1992	60	IVP (*n* = 30): 2 g of Cefmetazole administered by IVP IVI (*n* = 30): 2 g of Cefmetazole administered by IVI	No major adverse reactions were noted in either group. Phlebitis did not occur with either method of administration.	[26]

**Table 4:** PK outcomes of IVP and IVI.

Author	Year	No.	Groups	PK Outcomes	Ref.
Vanessa P. Ho et al.	2012	15	IVP (*n* = 10): 2 g of cefazolin administered by IVP IVI (*n* = 5): 2 g of cefazolin administered by IVI	C_30min_: 140.6 ± 62.9 μg/mL (IVP); 165.1 ± 38.5 μg/mL (IVI). T_1/2_: 2.3 ± 0.4 h (IVP), 2.9 ± 1.1 h (IVI).	[24]

**Table 5:** PK/PD-derived surrogate outcomes of IVP and IVI.

Author	Year	No.	Groups	Efficacy Outcomes	Ref.
Vanessa P. Ho et al.	2012	15	IVP (*n* = 10): 2 g of cefazolin administered by IVP IVI (*n* = 5): 2 g of cefazolin administered by IVI	fT > MIC: 3.6 h (IVP); 3.4 h (IVI) Protective duration: 5.1 h (IVP); 4.8 h (IVI).	[24]

**Table 6:** Economic outcomes of IVP and IVI.

Author	Year	No.	Groups	Economic Outcomes	Ref.
J C Garrelts et al.	1992	60	IVP (*n* = 30): 2 g of Cefmetazole administered by IVP IVI (*n* = 30): 2 g of Cefmetazole administered by IVI	Time efficiency: Pharmacy preparation time was shorter with IVP compared to IV infusion. Nursing administration time was shorter with IVP. Labor cost savings: Estimated cost avoidance: US $0.60 per dose due to reduced preparation and administration time. Material cost savings: US $3.25 per dose saved by eliminating the need for minibags and IV tubing with IVP administration. Institutional impact: Extrapolated total cost avoidance: approximately US $184,000 per year.	[26]

## Data Availability

No new data were created or analyzed in this study. Data sharing is not applicable to this article.

## Supplementary Materials

The following supporting information can be downloaded at: https://www.mdpi.com/article/10.3390/antibiotics15070643/s1, Table S1: Detailed search strategy and results (accessed on 22 February 2026); Table S2: Detailed search strategy of gray literature (accessed on 22 February 2026).

## Abbreviations

The following abbreviations are used in this manuscript:

fT > MIC	Free drug concentration above the minimum inhibitory concentration
SSI	Surgical site infections
IVI	Intravenous Infusion
IVP	Intravenous Push
PCC	Population, Concept, Context
MIC	Minimum Inhibitory Concentration
PK/PD	Pharmacokinetics/Pharmacodynamics
SOPs	Standard Operating Procedures
SWFI	Sterile Water for Injection
