# Supplementary material for: Administration Routes for Perioperative Prophylactic Antibiotics: A Scoping Review of Intravenous Push Versus Infusion
Source: Antibiotics (Basel). 2026 Jun 27;15(7):643. doi: 10.3390/antibiotics15070643 (PMC13405738; doi:10.3390/antibiotics15070643)
Supplement: Supplementary file 1 [file antibiotics-15-00643-s001.zip › antibiotics-4307951-supplementary.pdf]

**Supplementary Materials for**

*Review*

# Administration Routes for Perioperative Prophylactic Antibiotics: A Scoping Review of Intravenous Push versus Infusion

**Canyu Yang[1], Shuhua Deng[2], Yuan Wei[3], Yuxi Xia[4], Xiaoning Yuan[5], Ning Shen[6], Li Yang[1], Rongsheng Zhao[1], Yingqiu Ying[1,*]**

**Table S1. Detailed search strategy and results (Accessed on 2026/02/22)**

| Database | Query | Records found |
|---|---|---|
| PubMed | ((("intravenous push"[tiab] OR "iv push"[tiab] OR "bolus"[tiab] OR "intravenous infusion"[tiab] OR "iv infusion"[tiab] OR "piggyback"[tiab]) AND ("surgical site infection"[tiab] OR "surgical wound infection"[tiab] OR "prophylaxis"[tiab] OR "prophylactic"[tiab] OR "prevention"[tiab]) AND ("perioperative"[tiab] OR "surgical"[tiab] OR "surgery"[tiab]) AND ("cefazolin"[tiab] OR "cephalosporin"[tiab] OR "vancomycin"[tiab] OR "clindamycin"[tiab] OR "metronidazole"[tiab] OR "fluoroquinolone"[tiab] OR "penicillin"[tiab] OR "antibiotics"[tiab] OR "antimicrobial"[tiab])) AND ("effectiveness"[tiab] OR "safety"[tiab] OR "adverse events"[tiab] OR "adverse effects"[tiab] OR "harm"[tiab] OR "cost"[tiab] OR "economic"[tiab] OR "cost-effectiveness"[tiab])) NOT (retracted publication[pt]) | 35 |
| Embase | ('intravenous push' OR 'bolus injection'/exp OR 'intravenous infusion'/exp) AND ('surgical site infection'/exp OR 'surgical prophylaxis'/exp) AND ('perioperative period'/exp OR 'surgery'/exp) AND ('effectiveness'/exp OR 'safety'/exp OR 'adverse drug reaction'/exp OR 'cost effectiveness'/exp) AND 'antibiotic agent'/exp NOT 'retracted publication'/it | 71 |
| Web of Science | TS=("intravenous push" OR "iv push" OR "bolus" OR "intravenous infusion" OR "iv infusion" OR "piggyback") AND TS=("surgical site infection" OR "surgical wound infection" OR "prophylaxis" OR "prophylactic" OR "prevention") AND TS=("perioperative" OR "surgical" OR "surgery") AND TS=("cefazolin" OR "cephalosporin" OR "vancomycin" OR "clindamycin" OR "metronidazole" OR "fluoroquinolone" OR "penicillin" OR "antibiotics" OR "antimicrobial") AND TS=("effectiveness" OR "safety" OR "adverse events" OR "adverse effects" OR "harm" OR "cost" OR "economic" OR "cost-effectiveness") | 26 |

| | | |
|---|---|---|
| The Cochrane Library | #1 ("intravenous push" OR "iv push" OR "bolus" OR "intravenous infusion" OR "iv infusion" OR "piggyback"):ti,ab,kw<br>#2 ("surgical site infection" OR "surgical wound infection" OR "prophylaxis" OR "prophylactic" OR "prevention"):ti,ab,kw<br>#3 ("perioperative" OR "surgical" OR "surgery"):ti,ab,kw<br>#4 ("cefazolin" OR "cephalosporin" OR "vancomycin" OR "clindamycin" OR "metronidazole" OR "fluoroquinolone" OR "penicillin" OR "antibiotics" OR "antimicrobial"):ti,ab,kw<br>#5 ("effectiveness" OR "safety" OR "adverse events" OR "adverse effects" OR "harm" OR "cost" OR "economic" OR "cost-effectiveness"):ti,ab,kw<br>#6 #1 AND #2 AND #3 AND #4 AND #5 | 49 |

**Table S2. Detailed search strategy of grey literature(Accessed on 2026/02/22)**

| Type | Details |
|---|---|
| **Search Engine** | Google |
| **Search Terms** | Surgical Antibiotic Prophylaxis Administration guideline OR practice |
| **Search strategy** | To ensure the breadth of the search, the first 150 results (sorted by relevance) were screened for each search string. |
| **Types of literature identified** | Clinical practice guidelines, hospital internal protocols |
| **Records found** | 104 |
